# Tendon Vasculature in Health and Disease

**DOI:** 10.3389/fphys.2015.00330

**Published:** 2015-11-18

**Authors:** Herbert Tempfer, Andreas Traweger

**Affiliations:** ^1^Spinal Cord Injury and Tissue Regeneration Centre Salzburg, Institute of Tendon and Bone Regeneration, Paracelsus Medical UniversitySalzburg, Austria; ^2^Austrian Cluster for Tissue RegenerationVienna, Austria

**Keywords:** tendon vasculature, tendon stem/progenitor cells, tendinopathy, lymphatics, tendon regeneration

## Abstract

Tendons represent a bradytrophic tissue which is poorly vascularized and, compared to bone or skin, heal poorly. Usually, a vascularized connective scar tissue with inferior functional properties forms at the injury site. Whether the increased vascularization is the root cause of tissue impairments such as loss of collagen fiber orientation, ectopic formation of bone, fat or cartilage, or is a consequence of these pathological changes remains unclear. This review provides an overview of the role of tendon vasculature in healthy and chronically diseased tendon tissue as well as its relevance for tendon repair. Further, the nature and the role of perivascular tendon stem/progenitor cells residing in the vascular niche will be discussed and compared to multipotent stromal cells in other tissues.

## Introduction

Tendons are fibrous bands of connective tissue which connect muscle to bone and are able to withstand tension. Besides the mere transmission of force, their physiologic role is also the storage and recovery of energy, made possible by specific biomechanical properties. Upon damage, either due to traumatic injury and/or chronic degeneration, the macromolecular structure is disturbed, resulting in inferior tissue quality. Unlike in other, highly vascularized tissues such as skin or bone, neovascularization following tendon injury is not necessarily a hallmark of functional tissue repair. Instead, it is associated with degeneration as healthy tendons generally are poorly vascularized with relatively few cells embedded in an abundant collagen matrix. The aim of this review is to provide an overview of our current understanding of tendon vasculature and its role in healthy and developing tendon as well as the involvement of the vascular bed in tendon de- and regeneration.

## Vasculature in intact tendons

In early medical literature tendons are described as “*virtually dead during life*” (Edwards, 1946), based on the fact that tendons are poorly vascularized and contain only few cells compared to other tissues. Even though the vascular and lymphatic network had been visualized by dye injections, tendons were considered to be “*non-viable cables*” (Peacock, 1959). Nevertheless, although tendons resemble a sparsely vascularized tissue type they generally harbor more vessels than commonly believed.

### Mechanisms and sources of vascularization

Generally, blood vessels emanate into tendons from the musculo—tendineous junction, from the bone insertion site, and the so called “paratenon,” a loose areolar gliding tissue surrounding non-synovial tendons (Peacock, 1959; Schmidt-Rohlfing et al., 1992; Ahmed et al., 1998; Kannus, 2000). The complexity of the vascular network also depends on whether tendons are sheathed, that is embedded in synovial tissue, or is unsheathed. In 1953, Brockis J.G. has shown that in the sheathed digital flexor tendons of the palm vessels only enter the tendon at few distinct sites, whereas in the distal part of the pals and in the forearm, where the tendon is surrounded by paratenon tissue, vessels pass through the tissue more frequently (Brockis, 1953). These two “types” of tendon were later on referred to as “avascular tendons” and “vascular tendons,” with major implications for the understanding of adhesion formation following surgical repair (see below; Chaplin, 1973). Nevertheless, even though tendons clearly are not “*non-viable cables*,” they are poorly vascularized and particularly the avascular superficial zones of sheathed tendons are mainly nourished by diffusion from the synovial sheath.

Generally, the number of supplying vascular branches significantly differs between various tendons. For example, the patellar ligament is supplied by a total of three arteries and by the anastomotic arch from the Hoffa fat pad (Pang et al., 2009), whereas the Achilles tendon is supplied both by the peroneal and the posterior tibial arteries (Schmidt-Rohlfing et al., 1992). As tendons naturally are moving tissues which are extended by mechanical load, also the vasculature must be compliant to being stretched. In vascular tendons, vessels form “curves” within their embedding endotenon tissue, a loose areolar intratendineous tissue surrounding individual fascicles (Kannus, 2000). Upon loading of the tendon the vessels are stretched accordingly (Brockis, 1953). An illustrative and well described example for the anatomy of tendon vascularization is the rotator cuff, a group of four muscles and the connected tendons moving and stabilizing the glenohumeral joint. Despite the fact that all four tendons serve a relatively similar function, the supraspinatus tendon is unique in terms of its vascular bed. In this tendon initially an avascular zone, referred to as the “critical zone,” usually located about 1 cm proximal from the bony insertion has been described (Lindblom, 1939). However, subsequent studies demonstrated the presence of a vascular bed and it was postulated that the filling of the blood vessels was dependent on the positioning of the arm (Rathbun and MacNab, 1970). Such relative avascular zones have also been described for other tendons, i.e., the Achilles tendon (Stein et al., 2000) or the patella tendon (Clancy et al., 1981). Clinically these zones, also referred to as the “watershed area” in the Achilles tendon, are often prone to inflammatory episodes, potentially resulting in a painful and chronic tendinopathy (Józsa and Kannus, 1997) and/or rupture of the tendon (Alfredson et al., 2001).

The role of the vasculature during tendon development and maturation is also still poorly defined. Peacock (1959) describes embryonic tendons to be “*supplied with a rich capillary network*,” by analysing images of an 8 month old human embryo. A study in postnatal, immature sheep describes a massive decline in both cellularity and vessel density in the tendon of the extrinsic flexor muscles of the fingers (*musculus flexor digitorum superficialis*) (Meller et al., 2009). In line with these findings, several studies point out the (relative) decrease of cell density during tendon maturation (Ippolito et al., 1980; Józsa and Kannus, 1997; Oryan and Shoushtari, 2008). Given the fact that very little turnover of the extracellular matrix occurs in human tendons after termination of linear growth after (~ 17–18 years of age), low vascular supply seems appropriate (Heinemeier et al., 2013). For example, the half–life of collagen in mature equine tendons was calculated to be about 200 years (Thorpe et al., 2010).

**Figure 1:**
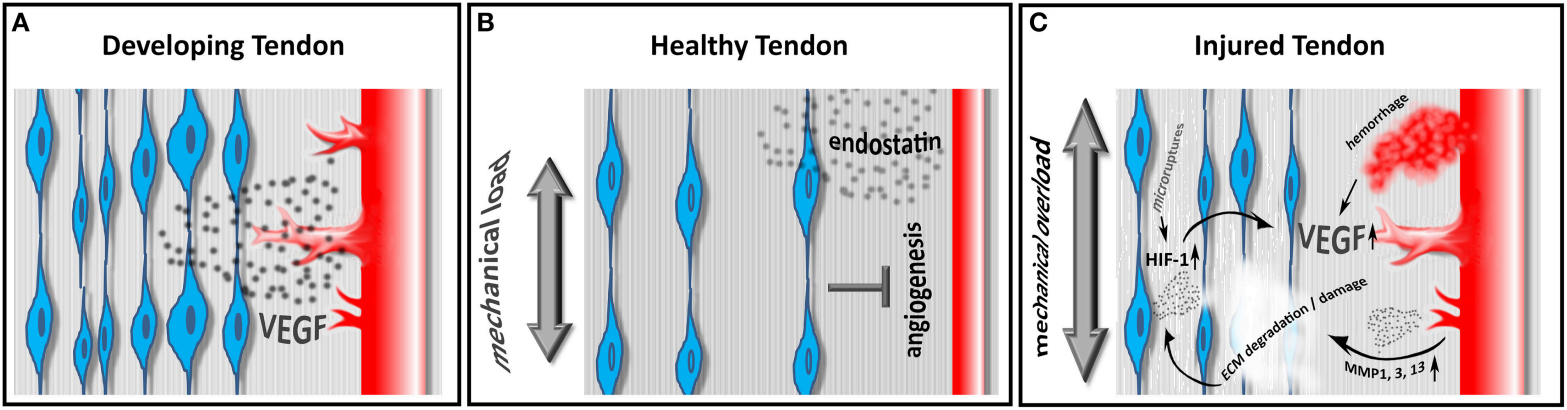
**Embryonic tendons show a high density of tendon cells producing VEGF, ultimately resulting in a pronounced angiogenic response in developing tendons (A)**. In healthy adult tendons the relative cell density decreases and tenocytes produce the antiangiogenic factor endostatin in response to physiological mechanical load, thus limiting neo-angiogenesis **(B)**. In diseased tendons, tenocytes produce HIF-1 in response to mechanical overload and/ or hypoxia. HIF-1 in turn induces the expression of VEGF, promoting neoangiogenesis. Hemorrhage due to vascular injury also leads to an increase in VEGF and to the production of matrix metalloproteinases (MMPs), resulting in a further weakening and degradation of the tendon matrix **(C)**.

The maintenance of hypo- or avascularity certainly requires either the production of antiangiogenic factors or the inhibition of proangiogenic factors. Indeed both mechanisms have been described in e.g., the hypovascular zones of sheathed tendons (Pufe et al., 2005). The proangiogenic protein vascular endothelial growth factor (VEGF) is found to be highly expressed in cells from fetal and injured human tendons, however only low expression is evident in intact adult tendons (Pufe et al., 2001) (Figure 1A). The antiangiogenic factor endostatin, a proteolytic fragment of Collagen XVIII, is also involved in tendon vascularization. The distribution of endostatin in gliding tendons correlates with the grade of vascularization. Endostatin expression is strong in the gliding area and reduced in areas without pressure and the expression levels are described to be influenced by mechanical load (Pufe et al., 2003) (Figure 1B). Taken together, our understanding of the molecular machinery controlling the complex vascularization process in tendons however remains fragmentary.

### Lymphatic drainage of tendons

So far, the lymphatic drainage of intact tendons has gained very little attention. Early literature reports lymphatic vessels to be associated with blood vessels in calf tendons as demonstrated by injection of India ink (Edwards, 1946). However, by now it is commonly accepted that the identification of lymph vessels simply by morphologic parameters is insufficient and even by means of immunohistochemistry it is challenging to separate blood from lymph vessels. A minimum of three lymph associated markers is recommended to distinguish lymph from blood vessels (Schroedl et al., 2014). Lymphatics play a crucial role in tissue repair mechanisms, due to injury or inflammatory processes and it has long been known that lymph vessels proliferate during inflammation. However, the role of lymphatic vessels in tendon disease has been neglected so far. To our knowledge there is no literature available on the lymphatic drainage in common tendon disorders such as tendinopathy, calcific tendinitis, or chronic tendon inflammation due to mechanic overuse. Recently, we have shown that intact rat Achilles tendons are void of lymphatics, which start to grow into the tendon repair tissue upon injury (Tempfer et al., 2015). Whether lymphatic ingrowth is a cause for impaired tissue quality and scar formation or merely a side effect requires further investigation. This may pave the way for future attempts to target lymphatic vessels to improve tendon regeneration, as it is successfully performed in other, non-musculoskeletal diseases, such as corneal and ocular surface inflammation (Bock et al., 2013).

### Tendon vessels as a niche for stem/ progenitor cells

As with many other tissues, vertebrate tendons harbor a population of stem/ progenitor cells (Salingcarnboriboon et al., 2003; Bi et al., 2007; Tempfer et al., 2009). These cells display mesenchymal stem cell (MSC)—like properties, displaying plastic adherence and a differentiation potential toward the osteoblast, adipocyte, and chondrocyte lineage. Further, they express classical MSC—associated surface markers such as Stro-1, CD44, CD90, and CD146 (Salingcarnboriboon et al., 2003; Bi et al., 2007; Rui et al., 2013) and reside within a fibromodulin- and biglycan-rich niche (Bi et al., 2007). We have shown that perivascular cells of the human supraspinatus tendon harbor a population of cells expressing both tendon- and stem cell associated markers (Tempfer et al., 2009). This is well in line with the finding that perivascular cells derived from a variety of tissues, such as skeletal muscle, pancreas, adipose tissue, and placenta show MSC–properties (Crisan et al., 2008). Interestingly, Mienaltowski et al. (2013) describe the presence of two distinct stem/progenitor cell populations within tendons, either located in the paratenon or the tendon proper. Both cell sources were negative for the perivascular surface marker CD133 and a differential expression pattern for the vascular marker endomucin as well as for tenomodulin and scleraxis. These data suggest that different stem/progenitor cell populations exist within distinct niches at the tendon proper and peritenon and the stem/progenitor cells in the peritenon might be more vascular in origin.

Generally, the role of perivascular MSCs in regeneration remains a matter of debate. Caplan A.I. proposes these cells to be activated upon local injury and to act as immunomodulatory agents guiding inflammation and regeneration (Caplan, 2008). Particularly in tendon, the function of resident stem/ progenitor cells remains unclear. As tendons naturally only have a poor regenerative capacity, leading to inferior tissue quality following injury or chronic degeneration, the pool of endogenous stem/progenitor cells apparently fails to functionally restore the damaged tissue within the milieu of an injured tendon (Voleti et al., 2012). Actually, tendon stem/progenitor cells are suspected to contribute to tendon degeneration by differentiation toward the osteoblast/chondrocyte lineage (Magne and Bougault, 2015). In diseases such as calcific tendinitis or in the formation of bony enthesophytes in spondyloarthritis, ectopic bone is formed within tendons. As tendon stem/progenitor cells have an even higher potential to undergo osteogenic differentiation than bone marrow derived MSC (Bi et al., 2007), it is speculated that they at least contribute to the pathogenesis of calcific tendinitis (Rui et al., 2011). Taken together, the *in vivo* role of tendon stem cells remains largely unknown and they potentially contribute to both tendon homeostasis and tendon pathologies by direct cell differentiation and/or production of trophic factors.

Nevertheless, transplantation of bone-marrow stromal cells and tendon-derived stem/progenitor cells has been proven to be beneficial for the functional repair of tendon tissue in various animal models (reviewed by Docheva et al., 2015). Recently, Lee CH et al. have shown that a rare tendon resident population of perivascular cells expressing CD146 can be expanded and stimulated *in vivo* by connective tissue growth factor (CTGF) in order to regenerate a tendon defect in a rat model (Lee et al., 2015). However, in order to fully harness the regenerative capacity of tendon stem cells we need to gain further insight into the *in vivo* identity of these cells and how they are modulated by the local niche. So far, this remains experimentally challenging due to the lack of tendon-specific markers.

## Vasculature in tendon disease

### Tendon adhesion formation

Peritendinous adhesions often lead to significant functional impairment after tendon surgery. Particularly sheathed tendons, such as the flexor tendon of the hand, frequently lose their gliding capacity after surgical repair, with a prevalence of ~ 4% being reported (Dy et al., 2012). In a rabbit study, three main factors have been identified, which in combination support the formation of adhesions: (i) suture of the partially damaged tendon, (ii) excision of the synovial sheath, and (iii) immobilization. If only one of these factors is avoided, adhesion formation can be significantly reduced (Matthews and Richards, 1976).

As nutrition of sheathed tendons is mainly provided by diffusion from the synovial membrane, the local loss of this tissue combined with a fibrin clot on the avascular outer layer of the tendon causes invasion of microvessels resulting in the formation of fibrous adhesions (Pennington, 1979). More recently, tendon adhesion formation using a mouse model for flexor tendon injury has been demonstrated to follow a typical wound healing response, with overlapping phases of inflammation, vessel ingrowth, and an increase in apoptotic cells over a follow-up time-period of 120 days (Wong et al., 2009).

Attempts to block adhesion formation by merely “wrapping” the tendon with organic or inorganic materials failed as the tendon proper became necrotic in many cases, indicating the importance of vascular supply (Weckesser and Shaw, 1949; Chaplin, 1973). Current research-based strategies include the use of multilayer membranes loaded with non-steroidal anti-inflammatory drugs (NSAIDs) to prevent fibrosis, mimicking the synovial membrane (Jiang et al., 2015) as well as the implantation of bioengineered synovia—like membranes (Baymurat et al., 2015).

### Vasculature in achilles tendinopathy

Tendinopathy is a painful, chronic disease commonly affecting various tendons such as the Achilles tendon or the tendons of the lateral elbow (“Tennis elbow”). As Achilles tendinopathy (AT) is the most frequent and best studied form of this disease, we will focus on this particular tendon. AT often affects people with high levels of sports activities. For example, 52% of elite long-distance runners are at risk for sustaining an Achilles tendon injury during their career (Kujala et al., 2014). AT is characterized by pain in the tendon during initial loading, subsiding with continued activity; as the condition becomes chronic, pain can be persistent. Overuse is considered to be the underlying cause; however the etiology and pathogenesis have not yet been fully clarified. Similarly, the source of the pain and the underlying mechanisms of pain remain unclear. Histologically, matrix disruption is commonly observed in AT, but is not necessarily involved in the pathogenesis as it also occurs in asymptomatic tendons (Magnan et al., 2014). Neovascularization is commonly seen in AT, as shown by Doppler sonography (Ohberg et al., 2001; Zanetti et al., 2003) and along with the ingrowth of vessels, also innervation is enhanced in tendinopathic zones, which may be causative for the pain associated with AT (Alfredson et al., 2001).

As mentioned above, during tendon development, high levels of VEGF are expressed. Molecules that are developmentally regulated are often re-expressed during the disease state. Indeed, the expression of VEGF in degenerative and spontaneously ruptured Achilles tendons is detectable at high concentrations when compared with adult, healthy Achilles tendons (Pufe et al., 2001). *In vitro*, cyclic mechanical load induces the expression of VEGF and hypoxia inducible factor 1 (HIF-1) in a frequency dependent fashion, indicating this mechanism being involved in tendon cell response to overload (Petersen et al., 2006) (Figure 1C).

Some authors report AT to be a result of an inadequate repair process following microtrauma, i.e., due to overuse. Because of the lack of blood vessels within the mid portion of the tendon a neurogenic inflammatory process is activated to repair these microruptures. This neurogenic inflammation occurs in the tissue surrounding the Achilles tendon and matrix and induces the expression of metalloproteinases (MMPs) responsible for the degradation of extracellular matrix. Concomitantly, cytokines such as VEGF, epidermal growth factor (EGF), and platelet-derived growth factor (PDGF) are overexpressed. VEGF not only promotes angiogenesis, but also upregulates the expression of MMPs and downregulates tissue inhibitors of metalloproteinases (TIMP-3), further progressing the remodeling of the tendon tissue (van Sterkenburg and van Dijk, 2011).

Tendon stem/ progenitor cells are also suspected to contribute to the pathophysiology of tendinopathies. In a rabbit tendinopathy model it was shown that tendon stem/ progenitor cells display an altered cell fate *in vitro*. They proliferate less and have a greater potential do undergo osteogenic and chondrogenic differentiation (Rui et al., 2011). Indeed, mineralization processes are also found in human AT and patella tendinopathy. One study on human tendinopathic tissue demonstrated that some mineralized deposits in Achilles and patella tendons are formed by a process resembling endochondral ossification, with bone formation and remodeling mediated by populations of osteoblasts and osteoclasts (Fenwick et al., 2002).

Regarding treatment strategies for AT, eccentric loading (e.g., muscle movements leading to muscle and tendon elongation) has shown to be a safe, cheap, and effective method to reduce pain and to improve tendon structure (Beyer et al., 2015). Interestingly, this method also reduces the number of neovessels in the affected area, which is considered to be causative for the beneficial outcome (Ohberg and Alfredson, 2004). Similarly, a combination of cryotherapy and compression of the tendinopathic area was shown to be an effective treatment, leading to a significant reduction of tendon blood flow (Knobloch et al., 2006). Also the use of topical nitroglycerin and low level laser irradiation are discussed to exert their positive effects by affecting tendon microcirculation. Nitroglycerin is a vasodilator and the positive effects reported are likely due to improved clearance of metabolic products, whereas low level laser irradiation may cause microthrombosis and/ or partial destruction of neovessels. However, for both therapies the underlying mechanisms of action remain poorly understood (Knobloch, 2008).

Another area often affected is the adult enthesis organ that connects tendons with bone, allowing the transmission of force from muscle to bone. The Achilles enthesis is frequently affected by non-inflammatory enthesopathies due to overuse or microtraumas and inflammation may occur resulting in major pain and disability. However, the underlying intrinsic and extrinsic factors also remain poorly understood.

Generally, mineralized and non-mineralized entheses can be differentiated. The Achilles tendon enthesis organ is mineralized and four zones can be distinguished: Zone 1 is built of dense fibrous connective tissue (the tendon proper), and the extracellular matrix (ECM) is mainly composed of collagen type I and III. Zone 2 consists of non-mineralized fibrocartilage and fibrochondrocytes. Here the ECM is formed by aggrecan and the collagen types I, II, and III. Zone 3 is formed of mineralized fibrocartilage with fibrochondrocytes, the predominant collagen being Col II next to I and X as well as calcium phosphate crystals. Between Zone 2 and Zone 3 the so called tidemark forms the boundary between soft, non-mineralized, and hard, mineralized tissue. Zone 4 finally is made up of the bone itself (Benjamin and McGonagle, 2009; Apostolakos et al., 2014). This gradual transition from compliant soft tissue to rigid bone absorbs local stress concentrations minimizing the risk of injury. Further, it has been reported that there is no direct cellular communication between bone and tendon tissue and the fibrocartilage at the enthesis acts as a barrier between cells in the two tissues (Ralphs et al., 1998). Whereas osteocytes and tendon cells directly communicate via gap junctions, the fibrochondrocytes in the calcified fibrocartilage zone were shown to lack similar structures.

Other than tendon, in a healthy state the enthesis is avascular (Dörfl, 1969). The lack of blood vessels reflects the compressive forces to which fibrocartilage is subject: vessel lumina would be occluded by compression. However, intratendinous vessels can anastomose directly with those of the bone at fibrous entheses (Benjamin and McGonagle, 2001). Similar to tendon, in development the enthesis is vascularized. During the growing period, bone grows into the tendon by endochondral ossification, where fibrocartilage is replaced by bone. Generally, cartilage erosion must be preceded by vascular invasion, yet it remains unclear how the blood vessels in the fibrous zone regress as the fibrous tissue is replaced by fibrocartilage (Gao et al., 1996; Benjamin and McGonagle, 2001).

## Conclusions

Neovascularization is critical to tissue repair and wound healing. Therefore, strategies to enhance vascularization to promote regeneration are considered promising treatment modalities, i.e., the use of platelet rich plasma (PRP) to restore functional bone (Zhang et al., 2013) or skin (Kakudo et al., 2011). However, in acute or chronic tendon injuries hypervascularity often does not pave the way to functional recovery of the tissue. Therefore, to overcome the limited intrinsic regeneration capacity of tendon and to achieve scarless healing will most likely require a balanced manipulation of the angiogenic response in tendon tissue. For a variety of treatment methods, such as the use of PRP, the availability of clinical data is limited, due to heterogeneity in application (Khan and Bedi, 2015). In order to develop rational strategies to achieve a well-balanced angiogenic response following tendon injury, we need a thorough understanding of the molecular and cellular networks driving tendon vascularization and regeneration—a challenge for years to come.

## Author contributions

HT and AT wrote the article.

## Funding

This work was supported by the Lorenz Böhler Funds (Vienna, Austria) and by the Paracelsus Medical University Research Funds (Salzburg, Austria) and the Hermann and Marianne Straniak foundation (Sarnen, Switzerland).

### Conflict of interest statement

The authors declare that the research was conducted in the absence of any commercial or financial relationships that could be construed as a potential conflict of interest.
